# Loach-inspired V-microgrooves coupled with microbubbles for stable gas film and enhanced underwater drag reduction

**DOI:** 10.1039/d6ra05288f

**Published:** 2026-09-28

**Authors:** Na Zhang, Lei Chen, Ying Lv, Tianyi Zhang, Bin Jia, Bobin Wang, Cuihong Liu, Hongguang Cui, Huan Wang, Yan Xu, Mingjin Xin, Liyan Wu

**Affiliations:** a College of Engineering, Shenyang Agricultural University Shenyang 110866 China wly78528@syau.edu.cn

## Abstract

Frictional drag significantly increases energy consumption in underwater vehicles. Inspired by loach scales, we designed a biomimetic drag reduction surface integrating V-shaped microgrooves and microbubbles. Numerical simulations based on the population balance model (PBM) in fluent revealed that bubble diameter and volume fraction are critical factors, achieving maximum drag reduction efficiencies of 29.1% at a bubble diameter of 50 nm and 30.2% at a 1.2% volume fraction, with pressure drag reduction as the dominant contributor. A flexible superhydrophobic surface was fabricated *via* vacuum demolding and thermal treatment, exhibiting a static contact angle of 157° and stable gas-film formation. Flow-channel experiments demonstrated a drag reduction efficiency (DRE) of 30.92% at 4.43 m s^−1^. These findings reveal the cooperative effect of surface microstructures and microbubbles in sustaining the air–water interface, providing a feasible strategy for high-efficiency underwater drag reduction in practical applications.

## Abstract 2

Loach-inspired V-shaped microgrooves synergize with microbubbles to stabilize the air water interface on a flexible superhydrophobic surface, achieving 30.92% drag reduction at 4.43 m s^−1^.

## Graphical abstract

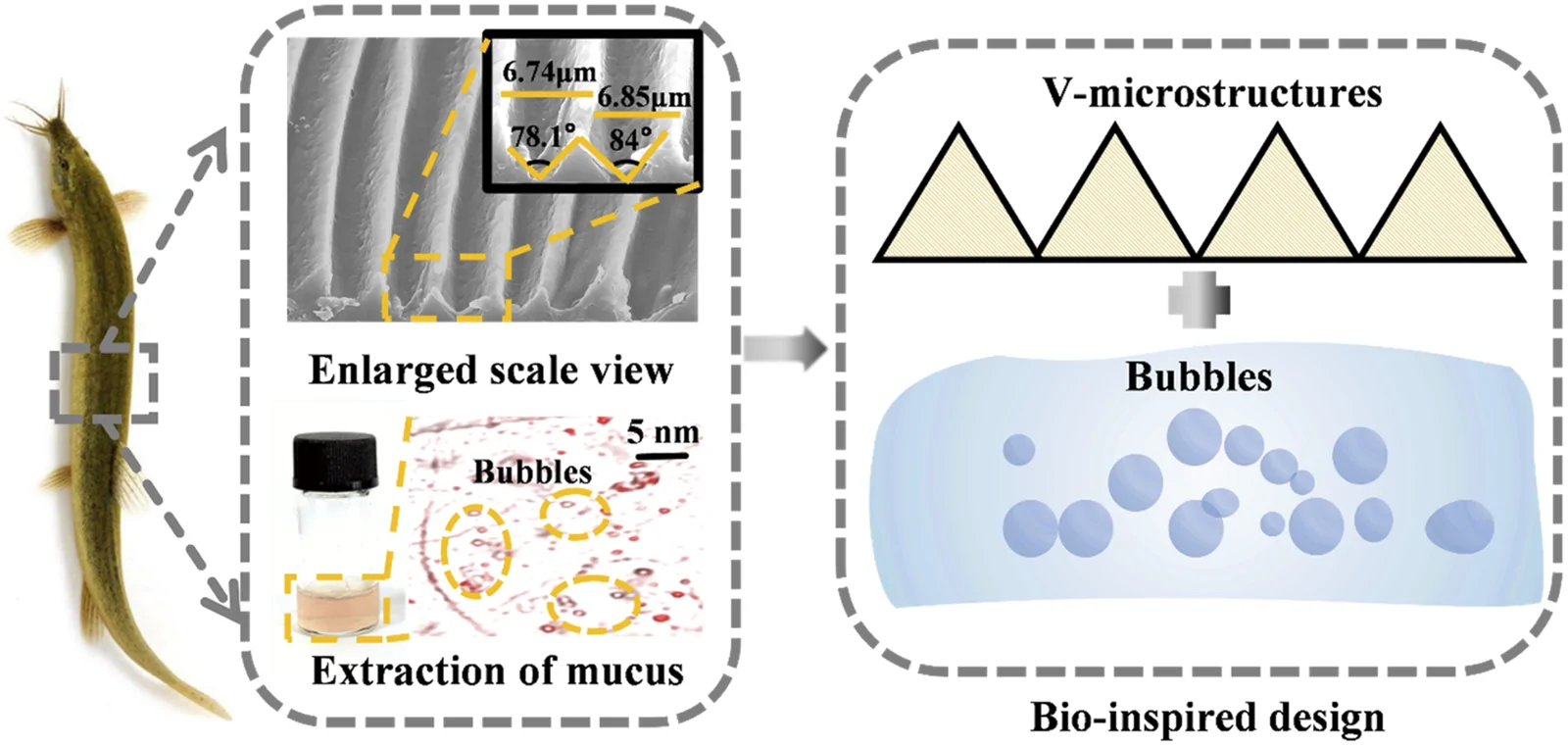

## 1. Introduction

The shipping industry experiences significant energy losses during high-speed navigation.^1–4^ A 10% reduction in surface drag can improve the speed and endurance of underwater vehicles by approximately 3.57%.^5^ Therefore, reducing the drag of underwater vehicles is one of the most effective approaches to reducing energy consumption and improving efficiency.

Existing drag-reduction strategies mainly include groove drag reduction,^6–8^ compliant wall drag reduction,^9–12^ microbubble drag reduction,^13–16^ polymer additive drag reduction,^17–20^ and superhydrophobic surface drag reduction.^21–23^ Groove structures can passively regulate near-wall flow without additional energy consumption; however, their drag-reduction performance strongly depends on geometric parameters and flow conditions. Compliant surfaces can modify near-wall turbulence through fluid–structure interactions, but their practical applications are restricted by fabrication complexity and long-term structural stability. Microbubble-based drag reduction reduces liquid–solid contact and modifies near-wall momentum transport, achieving high drag-reduction efficiency; however, maintaining a stable gas layer under high-speed and transient flow conditions remains challenging. Polymer additives can effectively suppress turbulent drag, whereas their continuous consumption and environmental compatibility limit long-term applications. Superhydrophobic surfaces reduce friction by maintaining a gas layer at the liquid–solid interface, but the stability of trapped gas layers under complex underwater conditions remains a major challenge. Therefore, integrating structural regulation with gas-phase retention may provide a promising strategy for achieving stable and efficient underwater drag reduction.

Recent studies have demonstrated that biomimetic microstructures with aerophilic or superhydrophobic characteristics can enhance bubble trapping and improve gas-layer stability. For example, inspired by Salvinia leaves, Hu Yue found that nanostructures could rapidly trap bubbles and form a stable gas layer.^24^ Inspired by the scale structure of butterfly wings,^25^ Chen found that hierarchical structures with regular cavities could effectively stabilize the gas–liquid interface. Nguyen investigated the effects of gas layers on drag and turbulent dynamics over superhydrophobic surfaces.^26^ Inspired by spider legs, Tan proposed underwater microcone nanostructures and demonstrated that increasing microcone height or decreasing pitch contributed to drag reduction.^27^ Using photolithography, Ou fabricated superhydrophobic surfaces with well-defined micropillar and microrib structures, achieving drag reduction of up to 40%.^28^ These studies demonstrate that rationally designed microstructures can promote gas retention and improve the performance of gas-assisted drag-reduction surfaces.

However, most previous studies have primarily focused on optimizing surface geometry to enhance gas retention. The synergistic interaction between surface morphology and gas-phase parameters, particularly the effects of bubble size and gas content on near-wall multiphase flow and drag-reduction behavior, remains insufficiently understood. The surface structures evolved in typical fish species, together with mucus on their body surfaces, exhibit remarkable drag-reduction characteristics^29^ and provide valuable inspiration for biomimetic applications. For example, Wu^30–32^ found that fabricated biomimetic loach-scale microstructures achieved a maximum DRE of 23% at 1.68 m s^−1^, while a grass carp-inspired crescent structure produced lubrication drag reduction through water collection. Inspired by the mucus secretion mechanism, Zhao designed a mucus-releasing structure, with a maximum DRE reaching 20.56%.^33^ Although these studies have advanced fish- and loach-inspired drag-reduction technologies, previous approaches have mainly emphasized surface morphology optimization or lubrication effects. The possible contribution of mucus-associated gas phases and their interaction with loach-inspired microgroove structures have received comparatively limited attention. In particular, the coupled influence of microgroove geometry and bubble characteristics on gas retention and drag reduction has not been systematically investigated.

In this study, the cross-sectional microstructure of loach scales and the bubbles in mucus were analyzed. Inspired by these biological characteristics and aiming to overcome the limitations of previous loach-inspired drag-reduction approaches, a synergistic drag-reduction strategy combining V-microgrooves and bubbles was proposed. Previous loach-inspired studies mainly focused on surface morphology optimization or mucus-inspired lubrication effects.^29–31^ In contrast, the present work introduces gas-phase regulation by coupling V-microgrooves with microbubbles, thereby extending loach-inspired drag reduction from single surface-structure optimization toward synergistic regulation of microstructure and gas-phase behavior. Numerical simulations based on an Euler–Euler framework coupled with the Population Balance Model (PBM) were conducted to investigate the influence of bubble parameters on multiphase drag-reduction behavior. Subsequently, a flexible superhydrophobic biomimetic specimen measuring 30 mm × 30 mm × 5 mm was fabricated using vacuum demolding and flame treatment. Finally, the surface wettability, gas-retention capability, and drag-reduction performance were experimentally evaluated through surface characterization and flow-channel experiments.

## 2. Materials and methods

### 2.1. Ethical statement

The loach was purchased from the market in Shenyang. The study was conducted according to the guidelines of the Declaration of Helsinki, and approved by the Institutional Review Board (or Ethics Committee) of Shenyang Agricultural University (protocol code 202108001 and date of approval 2021.3).

### 2.2. Characterization of the biomimetic prototype

The cross section of a loach scale, as shown in Fig. 1(a), exhibits V-microgrooves with a crest spacing of 7 µm and a groove angle of 80°. As shown in Fig. 1(b), the mucus contains bubbles of various sizes, with an average diameter of 1.12 µm.

**Fig. 1:**
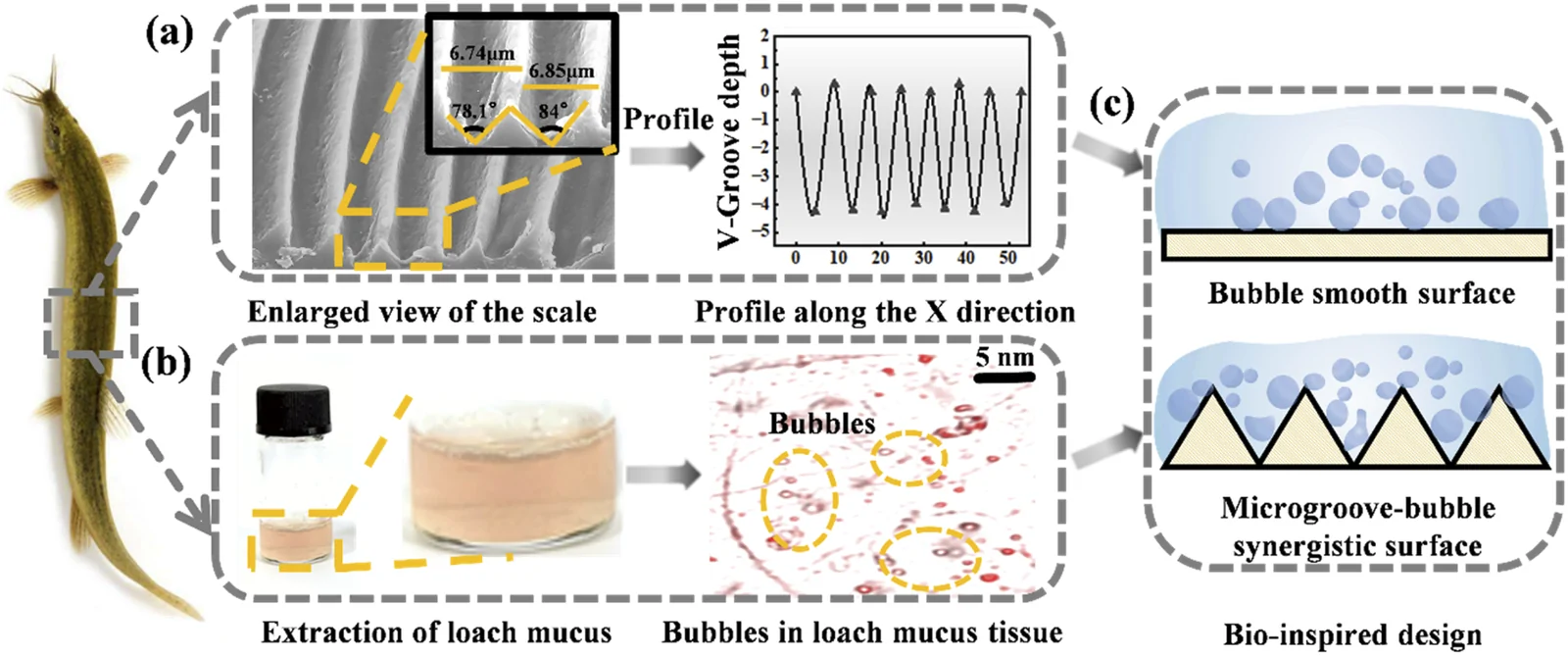
Bioinspired element identification and design. (a) SEM observation of the microstructures on the scales. (b) Microbubble in loach mucus. (c) Biomimetic model.

### 2.3. Fluid domain model and validation of mesh independence

Based on observations of the microscopic structures of loach scales, the 3D shown in Fig. 2 was established in this study, where *W* = 10 µm, *H* = 40 µm, and *L* = 100 µm. The V-microgroove was designed as an isosceles triangular groove with a base width of 7 µm and an included angle of 80°.

**Fig. 2:**
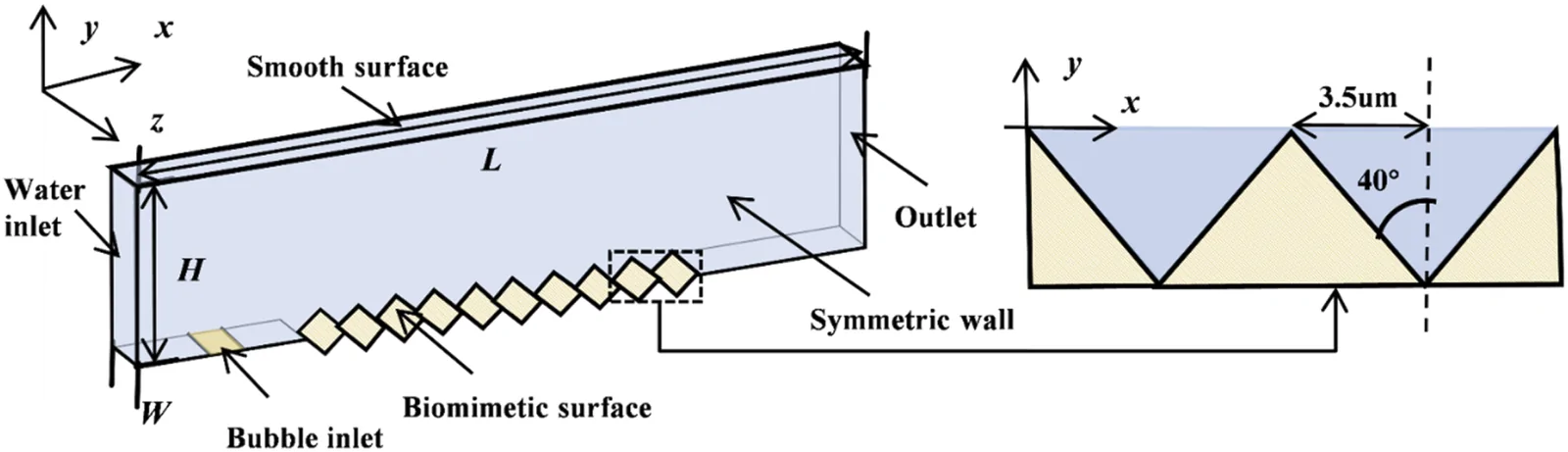
3D model of the biomimetic drag reduction structure.

A grid independence test was conducted for the 3D model by varying the number of nodes on one side of the biomimetic surface. When the relative error between two successive mesh refinements was less than 3%, the mesh density was considered satisfactory. The relative error is shown in Table 1. Considering both computational accuracy and cost, the final mesh count was determined to be 369 852.

**Table 1:** Validation of mesh independence and relative error

Experimental group	Grids number	Surface drag (N)	Relative error (%)
Q1	217 380	1.334 × 10^−6^	
Q2	369 852	1.3641 × 10^−6^	2.20%
Q3	599 724	1.3890 × 10^−6^	1.83%
Q4	846 516	1.4137 × 10^−6^	1.78%

### 2.4. Boundary conditions and computational methods

#### 2.4.1. Boundary conditions

Gravity was considered in the simulation. The present computational domain was constructed as a local near-wall region associated with the high-speed external flow environment considered for underwater applications. The standard *k*–*ε* model was employed to account for turbulent momentum transport associated with the external flow field, while the PBM was used to describe bubble breakup and coalescence. It should be noted that the Reynolds number based solely on the microscale dimensions of the computational domain is relatively low; therefore, the present model is intended primarily for comparative parametric analysis rather than prediction of microscale turbulence transition. The boundary conditions of the computational domain were set as follows: the import setting of water was defined as a velocity inlet with *V*_w_ = 5m s^−1^, and the import setting of bubble was also defined as a velocity inlet with *V*_b_ = 0.2m s^−1^, with both velocity directions perpendicular to the inlet boundary. The outlet was defined as a pressure outlet. Both the smooth surface and the biomimetic V-microgrooved surface were treated as no-slip walls with respect to the velocity boundary condition. Meanwhile, wall adhesion and contact-angle boundary conditions were introduced to account for the interaction between the gas–liquid interface and the solid surface. A static contact angle of 157° was assigned to the biomimetic V-microgrooved surface, consistent with the experimentally measured superhydrophobicity. Under these conditions, the near-wall gas distribution and gas retention within the V-microgrooves were governed by the combined effects of surface wettability, groove geometry, and multiphase transport. Therefore, although the solid wall itself remained no-slip, the retained gas phase formed a liquid–gas interfacial region with reduced direct liquid–solid contact and modified near-wall momentum transfer. The two side walls were treated as symmetry boundaries to represent an effectively extended surface in the spanwise direction.

#### 2.4.2. Computational methods

##### 2.4.2.1. Euler–euler model

In the Euler–Euler model, the interfacial area concentration equation was employed to capture the distribution of interfacial density at the bubble–liquid interface. Surface tension was taken into account in the calculations, and the Schiller-Naumann drag model was adopted. The drag coefficient was calculated according to eqn (1):1
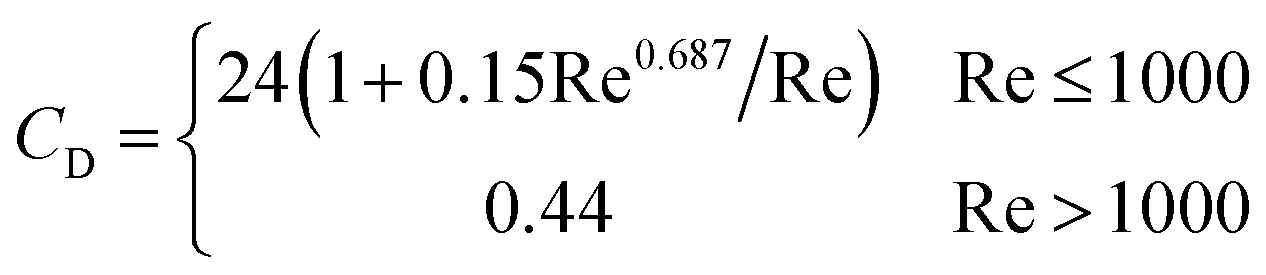
where *C*_D_ is the drag coefficient, and Re is the Reynolds number.

##### 2.4.2.2. Population balance model

The bubble population balance model, which is uniquely available in the Mixture and Eulerian frameworks for describing particle size evolution, can be used to investigate particle breakup and coalescence. In this study, the Luo model was selected for both breakup and coalescence.

The initial bubble size and the corresponding proportion were specified using a bin-based discretization method. Assuming spherical bubbles, the relationship between the bubble volume ratio and the bubble diameter ratio of two adjacent bins was determined according to eqn (2) and (3):

2 *V*_1_/*V*_2_ = *K*_v_π*d*_1_^3^/π*d*_2_^3^ = 2^*q*^

3 *d*_1_/*d*_2_ = (2^*q*^)^1/3^

where *K*_v_ denotes the particle volume coefficient, which is usually taken as π/6 for spherical particles, and *q* is the geometric ratio of particle volume. The volume fraction of each bin in the total volume was determined according to eqn (4):

4 *f*_*i*_ = *α*_*i*_/*α*

where *α*_*i*_ represents the sum of particle volumes in bin-*i*, *α* is the total volume of all particles, and *N*_*i*_ denotes the particle number concentration. *α*_*i*_ was determined by eqn (5):

5 *α*_*i*=_*K*_v_π*d*_*i*_^3^*N*_*i*_

To achieve a more accurate simulation of the bubble size distribution after breakup and coalescence, *q* = 2 and *N* = 6 were used in this study. By assuming a minimum bubble diameter of 20 nm, the size distribution of each bin was obtained as follows: *D*_0_ = 200 nm, *D*_1_ = 130 nm, *D*_2_ = 80 nm, *D*_3_ = 50 nm, *D*_4_ = 30 nm, *D*_5_ = 20 nm. These bubble sizes were prescribed numerical parameters for investigating the influence of bubble size evolution on multiphase drag-reduction behavior and should not be interpreted as a direct representation of the experimentally generated bubble-size distribution.

### 2.5 Fabrication of biomimetic V-microgrooved PDMS surface

A flexible biomimetic V-microgrooved PDMS surface was fabricated using a laser-machined silicon mold combined with a soft replication process. First, V-shaped microgroove structures were fabricated on a silicon wafer using laser micromachining. After processing, the silicon mold was cleaned with isopropanol to remove residual debris and contaminants generated during machining.

PDMS was prepared by thoroughly mixing the base elastomer and curing agent at a mass ratio of 10 : 1. The mixture was mechanically stirred and subsequently degassed under vacuum for 10 min to eliminate trapped air bubbles. The degassed PDMS precursor was then poured onto the silicon mold and thermally cured at 80 °C for 2 h. After curing, the elastomer was carefully peeled from the mold, producing a flexible PDMS replica containing V-shaped microgroove structures.

To further enhance the surface water repellency, the replicated PDMS surface was subjected to ethanol flame treatment. During treatment, the outer flame of an alcohol burner was uniformly moved across the sample surface in a transverse direction at an approximate scanning speed of 10 mm s^−1^ for 45–60 s. The flame treatment induced surface reconstruction and promoted the formation of hierarchical micro-nanoscale rough structures, thereby improving the hydrophobicity of the PDMS surface.

### 2.6 Surface wettability and water-repellency characterization

The wettability and dynamic water-repellent properties of the V-microgrooved PDMS surfaces were characterized through static water contact angle measurements and droplet bouncing experiments.

The static water contact angle was measured using a contact angle goniometer. A 6 µL droplet of deionized water was gently deposited onto the sample surface, and the contact angle was recorded after stabilization. For each specimen, measurements were performed at five randomly selected locations, and the average value was calculated.

Dynamic water repellency was evaluated through droplet bouncing experiments using a high-speed imaging system. A 6 µL water droplet was released from a height of 10 mm above the sample surface, and the impact, spreading, recoil, and bouncing processes were recorded. The droplet contact time and bouncing behavior were analyzed to evaluate the dynamic water-repellent performance of the fabricated surfaces.

### 2.7 Flow-channel drag-reduction experiment

The drag reduction performance of the biomimetic surfaces was evaluated using a water-channel experimental system. The flow velocity and pressure difference across the test section were monitored using a flow meter and a differential pressure sensor, respectively. The measurement accuracy of the pressure sensor was 0.2%.

The biomimetic specimen was horizontally mounted in the test section using a customized fixture, ensuring that the patterned surface was parallel to the incoming flow direction. Microbubbles were generated using a ZJC-NM series micro-nanobubble generator based on swirling counter-current mixing and gas–liquid shear mechanisms. During bubble generation, intense interfacial shear and pressure fluctuations induced by high-speed rotation promoted the formation of micro/nanobubbles. The microbubble generator was selected considering the stability of gas generation and compatibility with the flow-channel system. The generated bubble size distribution was characterized by nanoparticle tracking analysis (NTA). The measured mean diameter was 230.8 ± 3.4 nm, with a mode diameter of 125.4 ± 10.4 nm and a *D*_50_ value of 183.9 nm.

The generated bubbles were continuously introduced into the upstream region of the test section. The gas flow rate was maintained at 1 L min^−1^ during all experiments. Before data acquisition, the flow was stabilized for 2 min under each experimental condition. The pressure-drop data were then collected to calculate the drag reduction efficiency. Each experimental condition was repeated at least three times, and the averaged values were used for analysis. The detailed configuration of the water-channel system and experimental procedure were consistent with our previous studies.^29–31^6
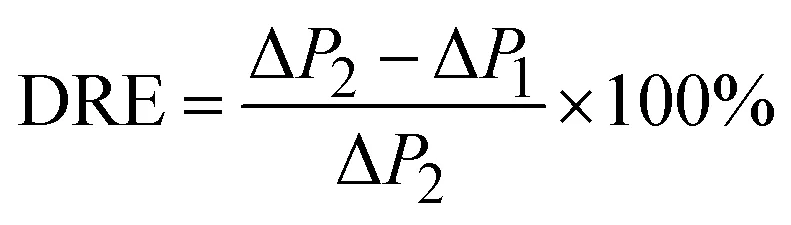
where Δ*P*_1_ and Δ*P*_2_ represent the pressure difference between the biomimetic sample and the smooth PDMS.

## 3. Results and discussion

### 3.1. Numerical simulation results and analysis

#### 3.1.1. Numerical simulation results

In this study, the biomimetic surface featured a V-microgrooved interface. When bubbles came into contact with this interface, they were prone to breakup, and the breakup rate increased with increasing bubble density and pressure.^34^ As two bubbles approach each other, a significant pressure difference is generated between them, thereby promoting bubble coalescence.^35^ As shown in Fig. 1(c), bubbles on the smooth surface are entrained by the water flow and swept away along the plate, whereas bubbles on the biomimetic surface undergo irregular motion over the grooved surface under the action of high speed flow. Meanwhile, the bubbles exert a squeezing effect on the surrounding fluid, which in turn exerts a reaction force on the bubbles,^36^ thereby altering the motion states of both the fluid and the bubbles. When bubbles pass over the groove crests, the abrupt change in surface geometry induces bubble breakup, leading to a more irregular distribution of the daughter bubbles.^37,38^ In addition, the rising velocity of bubbles in water is proportional to the square of their diameter.^39^ Owing to their extremely small size, micro-nano bubbles have an almost negligible rising velocity in water, which is conducive to their stable retention within the microstructures.

Numerical calculations were performed under the conditions of a water flow velocity of 5 m s^−1^, a bubble inlet velocity of 0.2 m s^−1^, a bubble volume fraction of 1%, and an initial bubble diameter of *D*_3_ = 50 nm. The drag on the biomimetic V-microgrooved surface (BV), bubble smooth surface (BS), and synergistic biomimetic surface (CB) were calculated, together with DRE and the total bubble volume (Table 2). CB was found to exhibit the best DRE and a certain bubble trapping capability. The drag reduction (DRE) is calculated as:

7 DRE = ((*F*_0_ − *F*)/*F*_0_) × 100%

where *F*_0_ is the drag on the smooth surface, and *F* is the drag on various types of surfaces.

**Table 2:** Numerical simulation results for different models

Surface type	Total drag (N)	Viscous drag (N)	Pressure drag (N)	DRE (%)	Bubble volume (×10^−17^ m^3^)
BV	1.6844 × 10^−6^	1.742 × 10^−7^	1.5102 × 10^−6^	9.23	—
BS	1.5796 × 10^−6^	1.5797 × 10^−6^	—	14.89	1.51
CB	1.3268 × 10^−6^	1.1551 × 10^−6^	1.7173 × 10^−7^	29.00	2.21

#### 3.1.2. Numerical simulation analysis

Numerical simulations were performed at a water flow velocity of 5 m s^−1^ and a bubble inlet velocity of 0.2 m s^−1^. Bubble diameters in the range of 30–100 nm (eight levels) and bubble volume fractions in the range of 0.5–1.2% (eight levels) were selected as variables for the calculations. The numerical simulation results are shown in Fig. 3(a and b). As the bubble diameter increased, the DRE first increased and then decreased, reaching a maximum of 29.1% at a bubble diameter of 50 nm. With increasing inlet bubble volume fraction, the calculated DRE gradually increased and reached a maximum of 30.2% at an inlet bubble volume fraction of 1.2% within the investigated range.

**Fig. 3:**
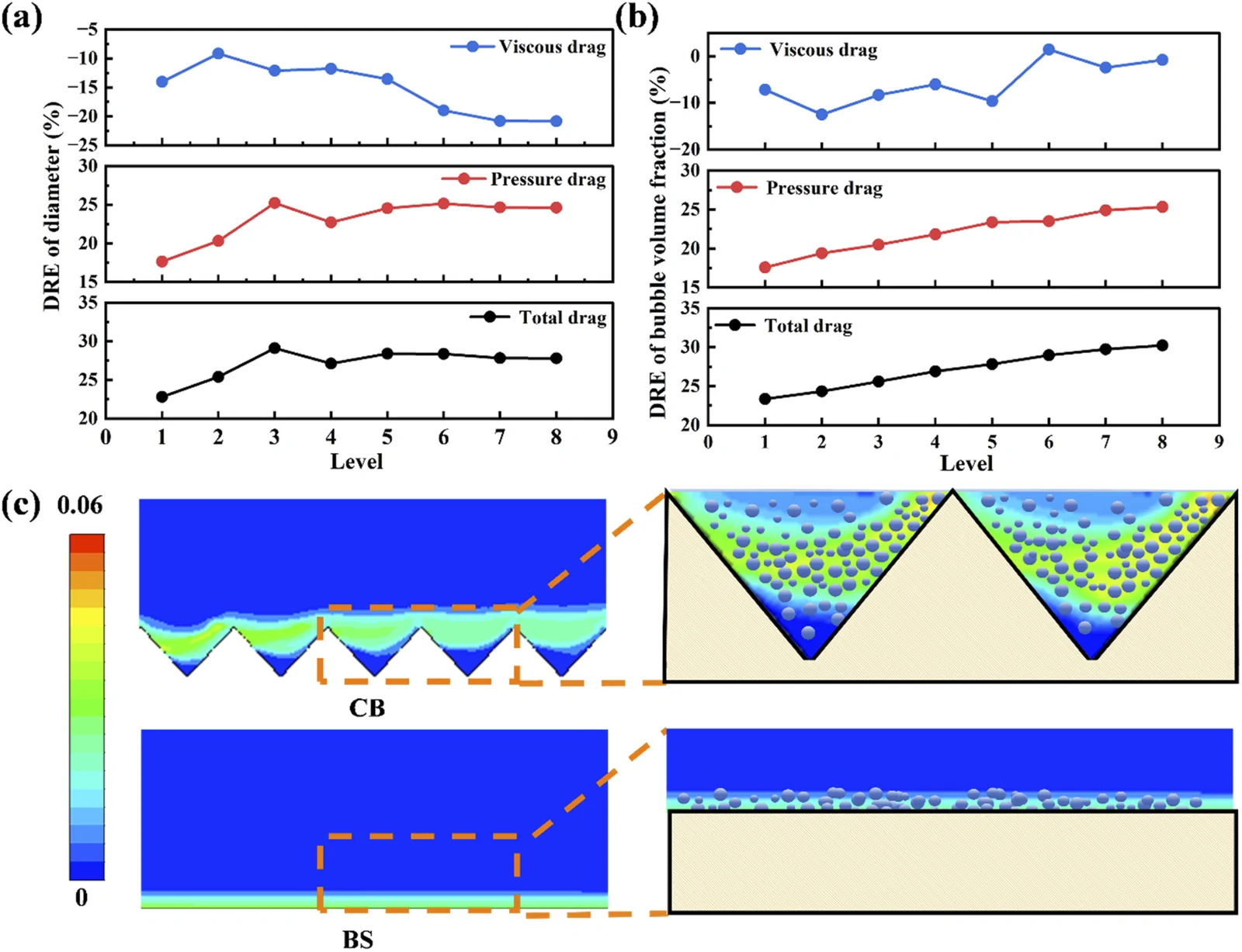
Numerical simulation results. (a) DRE of different diameters, (b) DRE of different bubble volume fractions, (c) characterization of the numerical simulation results.

This value represents a numerical optimum condition and does not directly correspond to the gas volume fraction in the experimental system. Under both conditions, the DRE of pressure drag remained positive, indicating that the introduction of bubbles reduced the overall drag primarily by decreasing pressure drag. Fig. 3(c) shows the bubble volume fraction contours of the CB and BS. On the CB, bubbles are trapped within the V-microgrooves either in the form of the gas film or by filling the V-microgrooves, forming a regular wavy interface above the V-microgrooves (Fig. 3(c)). This transforms the liquid–solid contact into a liquid–gas–solid interface, thereby reducing drag.

### 3.2. Drag reduction mechanism

#### 3.2.1. Analysis of turbulent dynamic viscosity

Fig. 4 presents the turbulent dynamic viscosity distributions for different inlet bubble diameters. Taking the area of a single V-microgrooves as one unit, the total area of the regions with high turbulent dynamic viscosity at each diameter was quantified numerically. In addition, as the inlet bubble diameter increases, the area of the high turbulent dynamic viscosity region on the CB gradually expands, which is consistent with the results calculated from eqn (8) and (9). When the inlet bubble diameter is smaller than 50 nm, the turbulent viscosity at the front of the CB is greater than that at the rear. This is because the velocity difference between the bubbles and the water is relatively large in the front region and gradually decreases along the flow direction. When the inlet bubble diameter is larger than 50 nm, the turbulent viscosity at the rear becomes greater than that at the front. This is because larger bubbles are less stable than smaller ones during motion and are more likely to coalesce into bubbles with even larger diameters, thereby increasing the turbulent viscosity in the rear region. Meanwhile, their interaction with the water flow induces stronger disturbances, resulting in a significant increase in turbulent viscosity at the rear of the CB. With increasing inlet bubble diameter, the turbulent viscosity at the front of the CB shows a decreasing trend, whereas that at the rear shows an increasing trend. Overall, the turbulent viscosity increases, leading to an increase in viscous drag.

8 *µ* = *ρ* × *v*

9 *v* = *C*_µ_*k*^2^/*ε* + 0.6*d*_a_*α*_a_|*U*_f_ − *U*_a_|

where *ρ* is the liquid density, *v* is the turbulent viscosity coefficient, and *C*_µ_ is taken as 0.09; *k* is the turbulent kinetic energy; *ε* is the turbulent dissipation rate; *d*_a_ is the bubble diameter; *α*_a_ is the air volume fraction; *U*_f_ is the water flow velocity; and *U*_a_ is the bubble velocity.

**Fig. 4:**
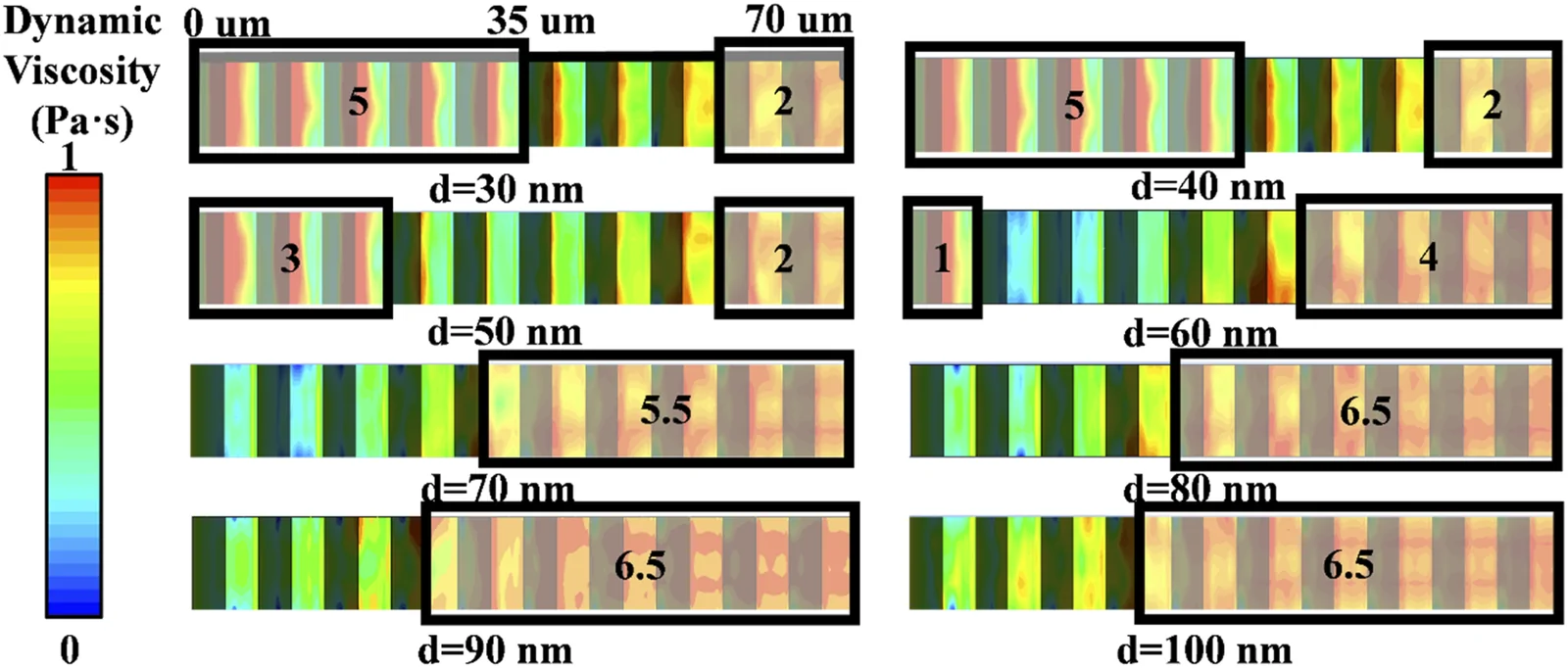
Turbulent dynamic viscosity distribution at different bubble diameters.

#### 3.2.2. Analysis of velocity field

The velocity contours and streamline distributions were analyzed for initial bubble diameters of 30 nm, 50 nm, 70 nm, and 90 nm. As shown in Fig. 5, when water flows through the V-microgrooves, small scale vortices are generated, significantly enhancing the transverse velocity fluctuations of the fluid. These vortices have a much greater impact on shear stress than the reduction in dynamic viscosity. As a result, the average wall shear stress on the CB surface is higher than that on the BV, contributing as an additional source of viscous drag. When the initial bubble diameter is less than 50 nm, the number of bubbles per unit volume increases, intensifying local momentum exchange and interfacial disturbances between the gas and liquid phases. Many small bubbles enter the V-microgrooves with the near wall shear flow, leading to the formation of numerous irregular small scale vortices within the V-microgrooves. When the initial bubble diameter exceeds 70 nm, the bubbles exhibit greater deformability and are prone to stretching, breakup, or coalescence with neighboring bubbles, enhancing flow fluctuations inside the V-microgrooves and near the V-microgrooves tops, resulting in a large number of unstable small scale vortex structures. Therefore, bubbles that are either too small or too large result in suboptimal drag reduction performance.

**Fig. 5:**
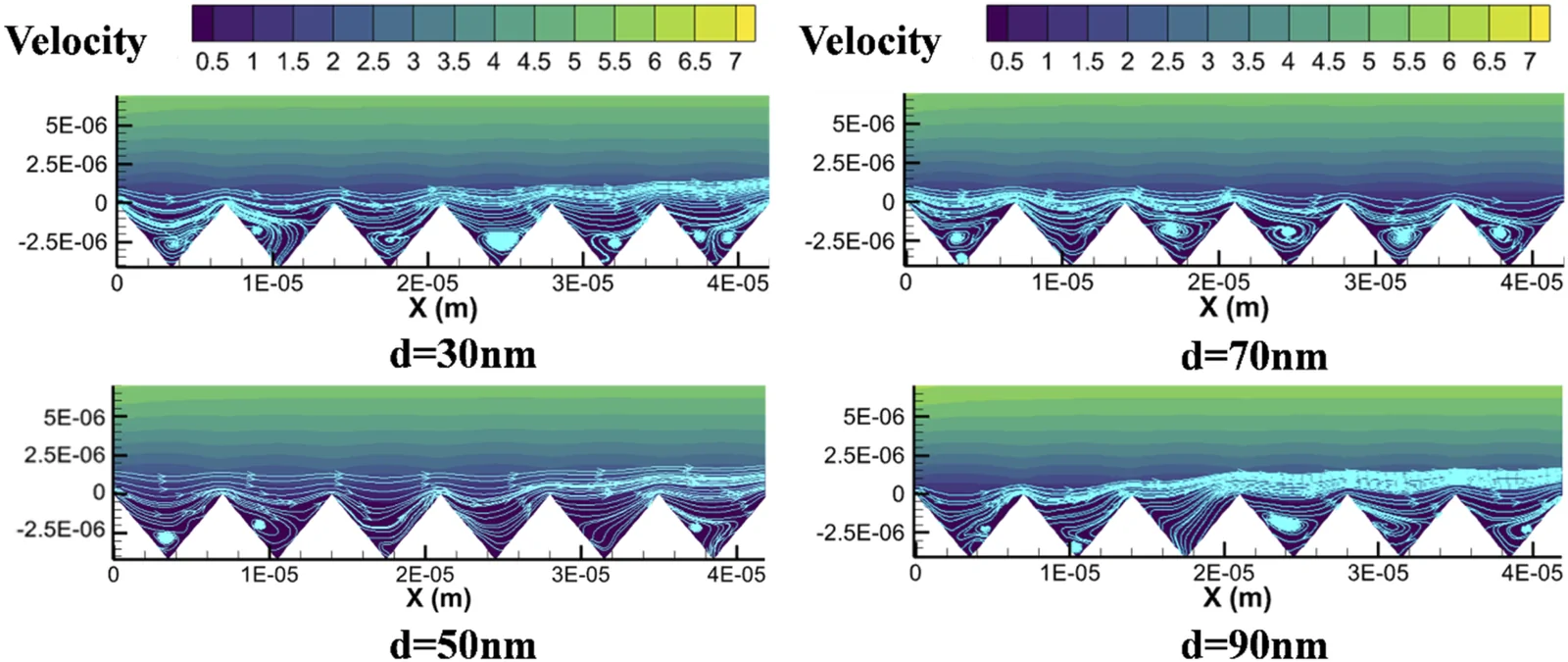
Velocity contours and streamlines at different bubble diameters.

#### 3.2.3. Analysis of bubble volume fraction

As shown in Fig. 6, the bubble volume fractions at *X* = 10.5 µm, 24.5 µm, 38.5 µm and 52.5 µm were compared among the synergistic models with different initial bubble diameters under the conditions of a water flow velocity of 5 m s^−1^, a bubble velocity of 0.2 m s^−1^, and an inlet bubble volume fraction of 1%. The bubbles are mainly distributed between the crests and troughs of the V-microgrooves, while the bubble volume fraction at the troughs is zero.^40^

**Fig. 6:**
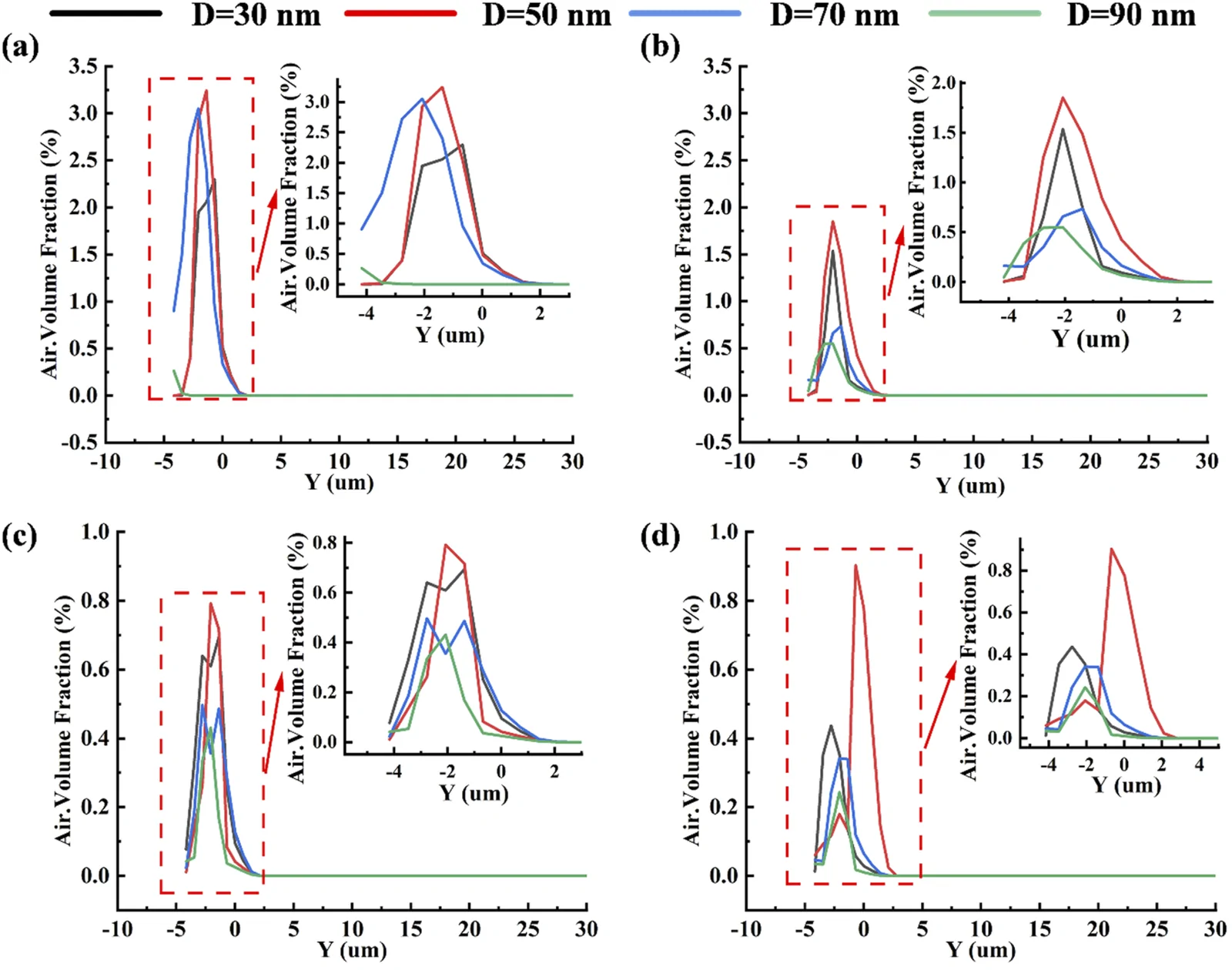
Bubble volume fraction at different cross sections. (a) *X* = 10.5 µm, (b) *X* = 24.5 µm, (c) *X* = 38.5 µm, (d) *X* = 52.5 µm.

This is because the bottom of the V-microgroove forms a sharp geometric corner with a near zero radius of curvature, creating a large pressure difference that prevents bubbles from moving into the groove bottom. After the calculations, the bubble volume showed a positive correlation with the DRE. The model with an initial bubble diameter of *D* = 50 nm exhibited the highest bubble volume fraction, and thus achieved the highest DRE. In addition, the gas film thickness (approximately 5 µm) is greater than the vertical depth of the V-microgrooves (4.2 µm), indicating that the bubble trapping capability of the V-microgrooves is not limited to the interior of the V-microgrooves, but can extend into the near wall region and form a continuous gas–liquid mixed layer.

### 3.3. Research on surface superhydrophobicity and drag reduction performance

#### 3.3.1. Fabrication of the biomimetic surface

Large area replication of flexible biomimetic specimens was achieved through vacuum demolding and heat treatment processes,^41^ as shown in Fig. 7(a). PDMS was selected as the demolding material.^42^ The demolded flexible biomimetic specimens inherently exhibited a certain degree of hydrophobicity. To further enhance the hydrophobicity of the specimen surface, the flexible V-microgrooved surface was subjected to flame treatment for 30–60 s. During this process, flame-induced surface modification occurred, resulting in the formation of micro/nanoscale hierarchical rough structures. The treated biomimetic specimens are shown in Fig. 7(b and d). Compared with the untreated surface, the treated surface exhibited increased surface roughness and enhanced structural hierarchy, which contributed to improved hydrophobicity and gas-retention capability. The obtained surface morphology was consistent with the designed biomimetic V-microgroove structure.

**Fig. 7:**
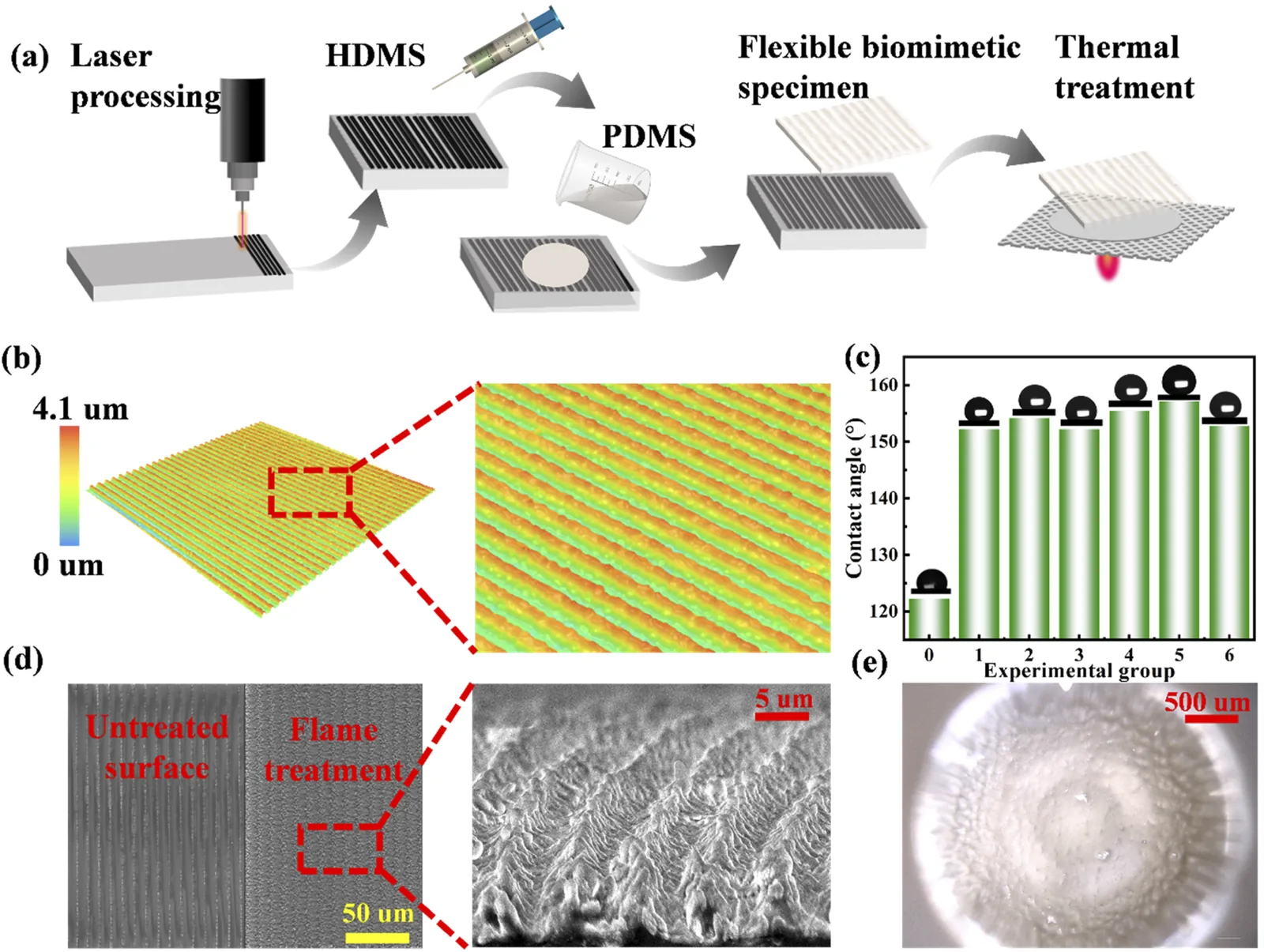
Fabrication and performance of the flexible biomimetic surface. (a) Fabrication process of the specimen, (b) morphology of the specimen observed by confocal microscopy, (c) contact angles of different specimens, (d) specimen interface after thermal treatment, (e) gas film under static water conditions.

#### 3.3.2. Static contact angle

To characterize the superhydrophobicity of the surface, static contact angle tests were conducted. As shown in Fig. 7(c), the sample labeled Test 0 corresponds to the original untreated specimen, which exhibited a static contact angle of 122.3°, indicating hydrophobicity. This behavior is mainly attributed to the low surface energy of the PDMS material and the V-microgrooves, causing the droplet to adopt a partial Cassie–Wenzel hybrid wetting state on the surface. The results for Tests 1–6 show that the droplets remained nearly spherical on the specimen surface, with contact angles reaching up to 157° and an average of 153.8°, indicating that the surface possesses superhydrophobicity.

#### 3.3.3. Static air cavity test

The surface of the flexible superhydrophobic biomimetic specimen exhibited a silvery reflective appearance (Fig. 7(e)).^43^ This is because the V-microgrooves, together with the superimposed nanoscale roughness, prevent the droplet from overcoming the capillary intrusion pressure and penetrating into the V-microgrooves, allowing bubbles to remain trapped within the structural gaps. When light passes from water (refractive index *n* ≈ 1.33) across the interface to air (refractive index *n* ≈ 1.00), part of the incident light is reflected and part is refracted, resulting in a silvery mirror appearance. This reflection effect is generally considered an important optical signature of a stable trapped gas film on superhydrophobic surfaces.

#### 3.3.4. Droplet bouncing test

On underwater vehicle surfaces, droplet bouncing behavior helps reduce fluid adhesion and the drag induced by attached droplets, thereby improving operational efficiency.^44–46^ The experimental results showed that the number of droplet bounces remained consistently at 14 ± 1.

Fig. 8 illustrates the complete dynamic bouncing process of a droplet on the superhydrophobic surface. During the impact stage, inertial forces dominate, causing the droplet to spread radially. At maximum spreading, inertial forces and surface tension reach a transient equilibrium. Subsequently, during the recoiling stage, surface tension becomes dominant, driving the droplet to retract and recover part of its kinetic energy. When the recovered kinetic energy exceeds the combined effects of gravity and interfacial adhesion, the droplet detaches from the surface, achieving complete bouncing.

**Fig. 8:**
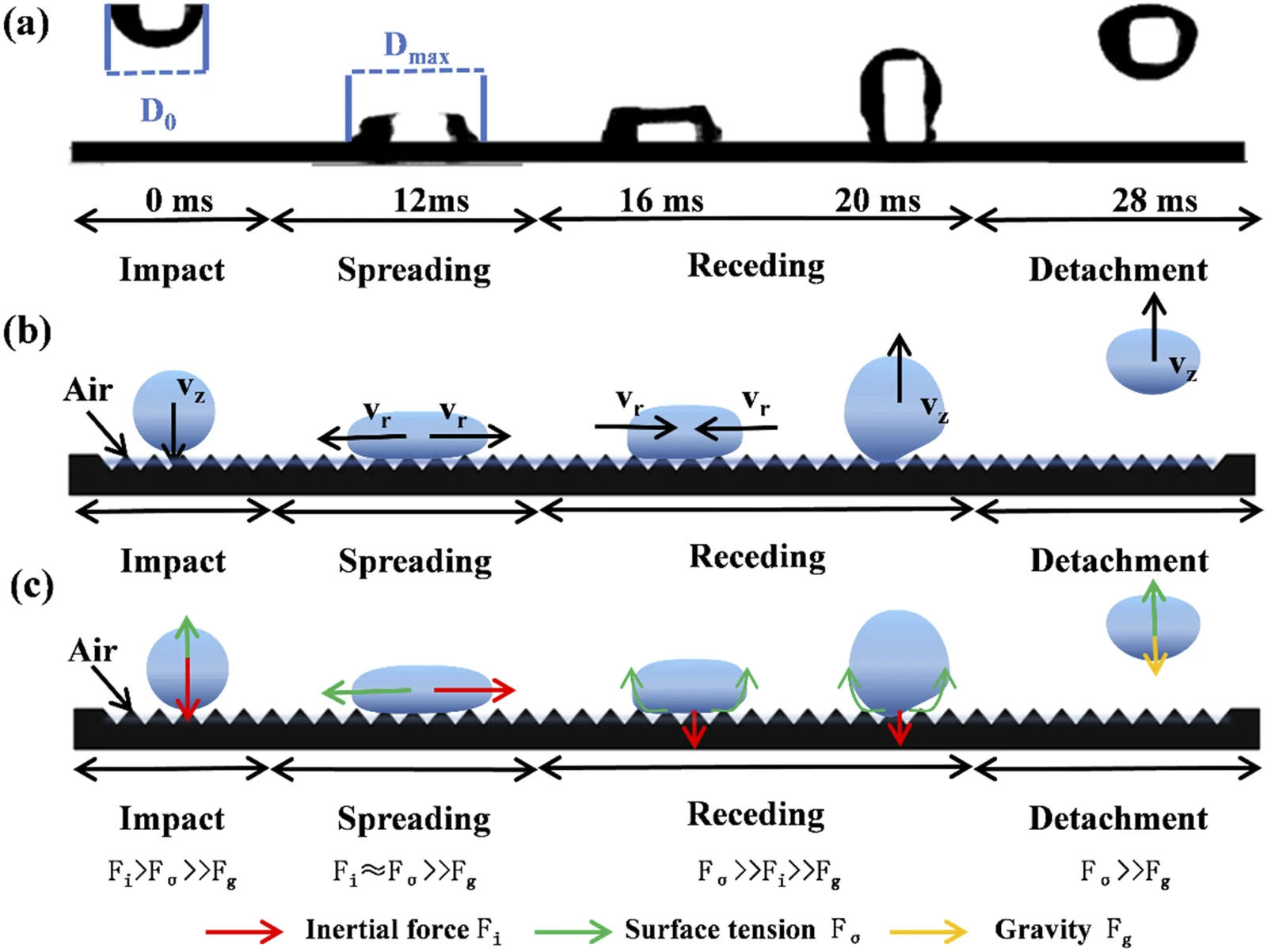
Droplet bouncing test. (a) Different stages of droplet bouncing, (b) velocity at each stage, (c) forces at each stage.

#### 3.3.5. Drag reduction performance

To further evaluate their drag reduction performance and durability under realistic conditions, flow-channel experiments were carried out in this study (Fig. 9 and 10(b)). The pressure drop of the gas–liquid two phase flow along the surface of the biomimetic specimen was measured to calculate the DRE. After the experiments, the tested specimens were further analyzed.

**Fig. 9:**
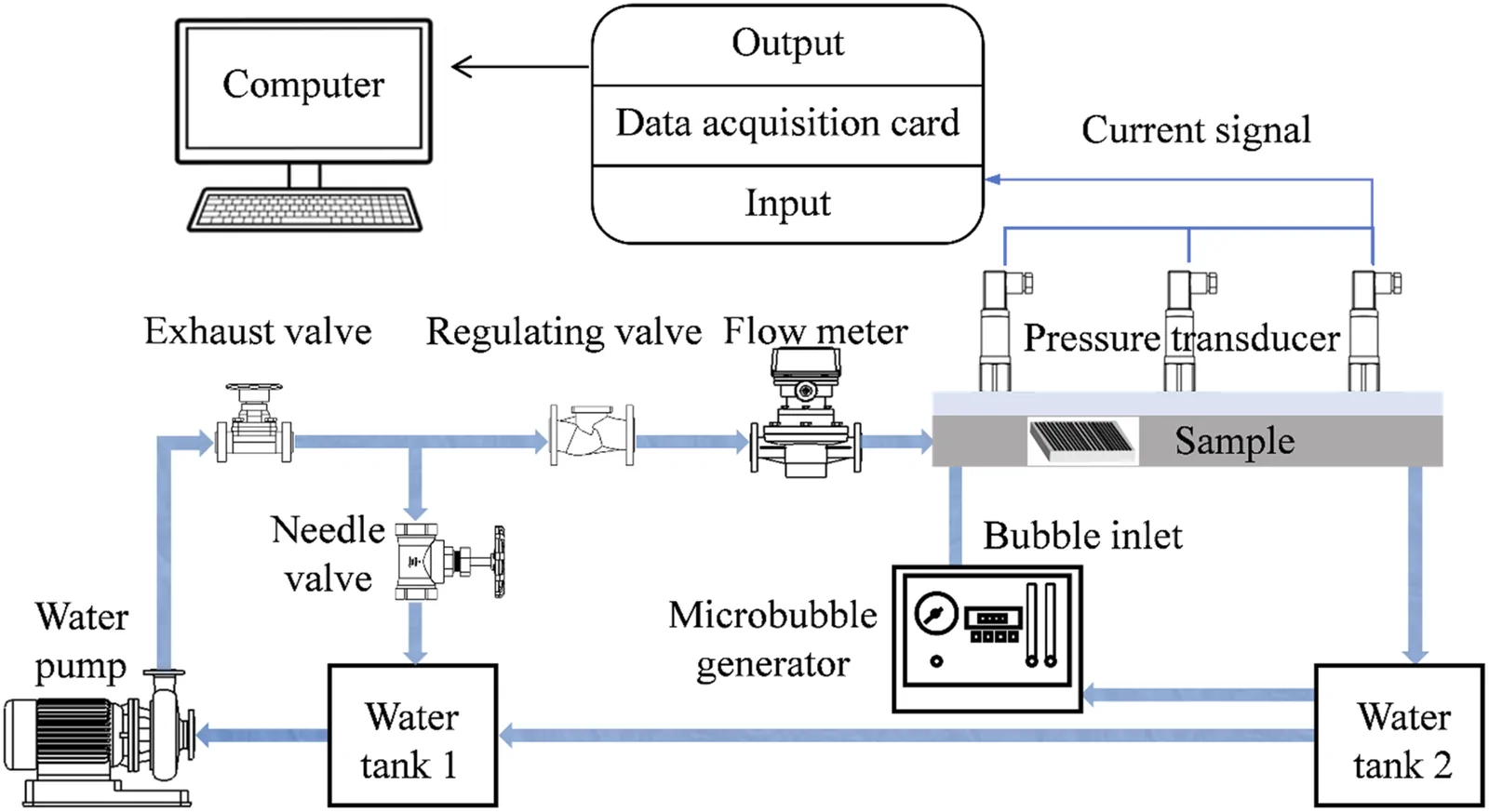
A simplified schematic diagram of the flow-channel setup.

**Fig. 10:**
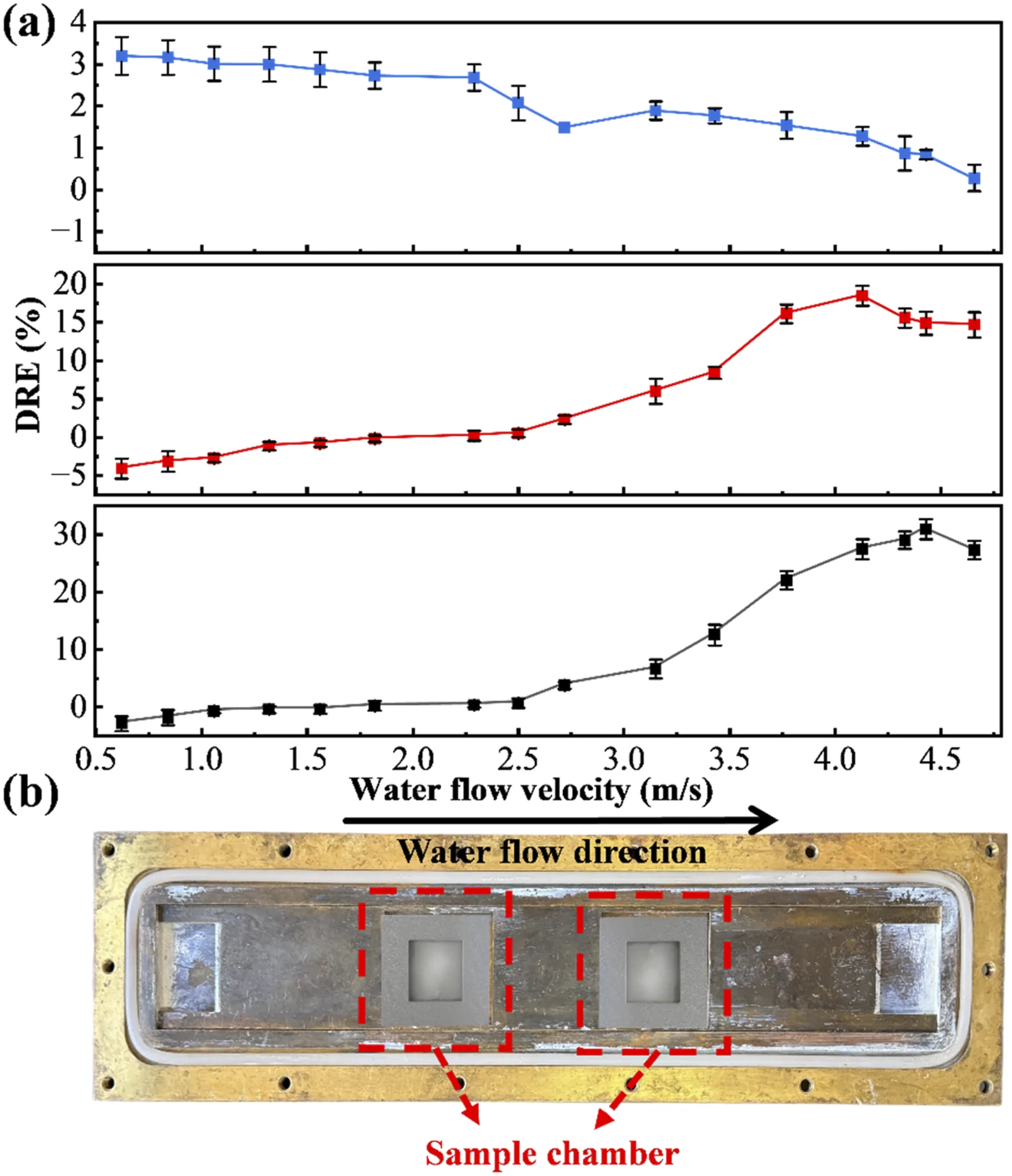
Flow channel experimental evaluation. (a) Photograph of the test section, (b) flow-channel experimental results.

As shown in Fig. 10(a), when the water flow velocity was below 1.56 m s^−1^, neither BV nor CB produced drag reduction; instead, both led to an increase in drag, indicating that under low speed conditions, the introduction of bubbles aggravates flow disturbances near the surface. When the water flow velocity exceeded 2.72 m s^−1^, the DRE ranked as CB > BS > BV. Specifically, at the water flow velocity of 0.62 m s^−1^, BV achieved the maximum DRE of 3.2%; at 4.13 m s^−1^, BS reached the maximum of 18.44%; and at 4.43 m s^−1^, CB attained the maximum DRE of 30.92%. It should be noted that the experimentally generated bubbles exhibited a broader submicron-scale size distribution than the prescribed bubble diameters used in the numerical parametric analysis. Therefore, the numerical and experimental results are not interpreted as a direct one-to-one validation of the specific optimum parameters. Rather, the simulations were used to reveal the relative effects of bubble diameter and gas volume fraction, whereas the flow-channel experiments were used to evaluate the practical drag-reduction performance of the coupled surface.

## 4. Conclusions

A loach-inspired synergistic drag-reduction strategy combining V-shaped microgrooves and microbubbles was proposed and investigated numerically and experimentally. Numerical results showed that bubble diameter and gas volume fraction significantly affect drag-reduction performance, with favorable performance obtained at a bubble diameter of 50 nm and a gas volume fraction of 1.2% within the investigated parameter range.

The fabricated flexible superhydrophobic biomimetic surface exhibited a static contact angle of 157° and effective gas-retention capability. Flow-channel experiments showed that the coupled V-microgroove–microbubble surface achieved a maximum drag reduction efficiency (DRE) of 30.92% at 4.43 m s^−1^, outperforming the groove-only and bubble-only surfaces.

Overall, the numerical and experimental results reveal the proposed synergistic drag-reduction strategy from complementary perspectives: the simulations clarify the influence of gas-phase parameters on drag-reduction performance, while the experiments demonstrate the practical drag-reduction capability of the coupled surface. This work extends loach-inspired drag reduction from single surface-morphology optimization toward microstructure-gas-phase synergistic regulation.

## Author contributions

Na Zhang and Lei Chen performed writing – original draft and methodology. Liyan Wu and Huan Wang performed conceptualization and writing – original draft. Hongguang Cui, Cuihong Liu, Bin Jia, and Bobin Wang performed formal analysis and writing – review & editing. Mingjin Xin, Yan Xu, Ying Lv, and Tianyi Zhang performed software and data curation. All authors have given approval to the final version of the manuscript.

## Conflicts of interest

The authors declare there is no conflict of interest.

## Data Availability

The data that support the findings of this study are available from the corresponding author upon reasonable request.
